# Der Umgang mit Notfallklassifikationen – Wo stehen wir? Ergebnisse einer deutschlandweiten Umfrage

**DOI:** 10.1007/s00101-021-00971-2

**Published:** 2021-05-18

**Authors:** A. Brosin, P. Kropp, D. A. Reuter, M. Janda

**Affiliations:** 1 Klinik und Poliklinik für Anästhesiologie und Intensivtherapie, Universitätsmedizin Rostock, Schillingallee 35, 18057 Rostock, Deutschland; 2 Institut für Medizinische Psychologie und Medizinische Soziologie, Universitätsmedizin Rostock, Gehlsheimer Str. 20, 18147 Rostock, Deutschland

**Keywords:** Krankenhausmanagement, Notfallmanagement, Notfallkategorie, OP-Management, Umfrage, Hospital management, Emergency management, Urgency category for emergency, Operating room management, Survey

## Abstract

**Hintergrund:**

Ziel der vorliegenden Studie ist eine aktuelle Standortbestimmung im Hinblick auf die Umsetzung der Empfehlungen zur Klassifikation von Notfalloperationen, welche von den Verbänden BDA/DGAI, BDC/DGCH und VOPM im Jahr 2016 veröffentlicht wurden.

**Methodik:**

In Anlehnung an die gemeinsamen Empfehlungen der Fachverbände wurden mithilfe eines Online-Fragebogens verschiedene organisatorische Aspekte der operativen Notfallversorgung untersucht. Hierzu wurden bundesweit OP-Manager/‑Koordinatoren an operativ tätigen Kliniken mit einer Mindestanzahl von 200 Betten befragt.

**Ergebnisse:**

An der Umfrage beteiligten sich 274 der 550 angeschriebenen Kliniken (49,8 %). Die Empfehlungen werden aktuell in 70,7 % der Häuser umgesetzt. Die Auffassung, dass die Notfallklassifizierung die zeitgerechte Notfallversorgung von Patienten verbessert, teilt eine Mehrheit von 78,2 % der OP-Verantwortlichen. 33,6 % sind allerdings auch der Ansicht, dass die definierten Zeitintervalle zur Umsetzung der Notfälle die Möglichkeit einer subjektiven Auslegung bieten. Zusätzliche hausinterne Empfehlungen zu den am häufigsten auftretenden Notfallindikationen würden 80,1 % als hilfreich erachten; in 39,1 % der Häuser sind diese bereits implementiert. 65,2 % der Krankenhäuser halten für die Versorgung von Notfällen keine zusätzliche Notfallkapazität vor, 30,1 % arbeiten hingegen mit definierten Konzepten zur Sicherstellung der bedarfsgerechten Verfügbarkeit von Saalkapazitäten.

**Schlussfolgerung:**

Die Empfehlungen zur Notfallklassifikation sind über alle Versorgungsstufen hinweg in der klinischen Realität Deutschlands angekommen und werden von der großen Mehrheit der OP-Verantwortlichen als hilfreiches Instrument in der OP-Koordination erachtet. Zusätzliche, indikationsbezogene Empfehlungen zur Klassifizierung der am häufigsten auftretenden Notfalleingriffe werden mehrheitlich befürwortet. Das Vorhalten eines definierten Notfallsaales ist entgegen bisherigen Annahmen in der deutschen Krankenhauslandschaft nahezu die Ausnahme.

**Zusatzmaterial online:**

Die Online-Version dieses Beitrags (10.1007/s00101-021-00971-2) enthält den zugrunde liegenden Fragebogen. Beitrag und Zusatzmaterial stehen Ihnen auf www.springermedizin.de zur Verfügung. Bitte geben Sie dort den Beitragstitel in die Suche ein, das Zusatzmaterial finden Sie beim Beitrag unter „Ergänzende Inhalte“.

## Abstract (en)

**Background:**

This study aimed to determine the current state of implementation of the recommendations for the classification of emergency surgery published in 2016 by the German societies of anesthesiology (BDA/DGAI), surgery (BDC/DGCH) and operating room management (VOPM).

**Methods:**

Based on these societies’ recommendations, various organizational issues were explored using an online questionnaire that was limited to German operating room (OR) managers and coordinators for hospitals that had surgical programs and at least 200 hospital beds.

**Results:**

A total of 550 hospitals were contacted and 274 participated in the survey (49.8%). Of these 70.7% reported that they had implemented the recommendations, and 15.2% were aware of the recommendations but did not consistently apply them. Of the participating OR managers and coordinators that had either implemented or were aware of the recommendations, 78.2% agreed that the standardized definition of medical emergencies led to improvements in emergency treatment but 33.6% stated that the defined response intervals for emergency categories induced a certain degree of subjectivity in categorizing emergencies. Additional in-house guidelines specifically for the most frequent surgeries were or would be welcomed by 80.1% of the respondents and 39.1% of the surveyed hospitals had already implemented such guidelines. Of the OR managers and coordinators, 62.9% were informed about their emergency volumes and 47.3% stated that they regularly assessed them. There was no dedicated capacity for emergency care in 65.2% of hospitals. Of the respondents 3.9% stated that a separate emergency OR was reserved with a freely available team, which, during core operating hours, could be used for interdisciplinary emergency care and 26.2% of hospitals considered the capacity required for emergency procedures when planning the OR program or determining OR capacities.

**Conclusion:**

The recommendations for classifying emergency operations are an essential and generally accepted control mechanism in OR coordination. They simplify interdisciplinary coordination and communication when dynamically incorporating emergency procedures into an OR program. Most OR managers and coordinators view the recommendations as improving the speed of action in emergency care. To support the adoption of emergency classifications within an organization it may be advisable to incorporate them into the OR statutes and integrate them within the hospital information systems. The majority of participants supported additional specifications based on medical indicators for classifying the most frequent emergency operations. Being cognizant of key metrics concerning in-house emergency volume represents a crucial basis for interdisciplinary OR management and emergency care integration. Contrary to common perception, blocking fixed OR capacities remains the exception. When establishing a concept to provide emergency capacity, it is advisable to align developments with demand calculations based on in-house figures and to emphasize interdisciplinary participation and consensus.

Mit dem Glossar perioperativer Prozesszeiten und Kennzahlen in der Version 2016 [4] wurde zwischen den Berufsverbänden Deutscher Anästhesisten (BDA) und Deutscher Chirurgen (BDC), der Deutschen Gesellschaft für Anästhesiologie und Intensivmedizin (DGAI), der Deutschen Gesellschaft für Chirurgie (DGCH) sowie dem Verband für OP-Management (VOPM) eine einheitliche Definition der medizinischen Dringlichkeit operativer Notfälle sowie der koordinativen Reaktion bei der Umsetzung im operativen Tagesgeschäft abgestimmt. Über die tatsächliche Implementierung dieser Vereinbarung im klinischen Alltag existieren bisher keine Daten. Anhand der vorliegenden Umfrage wurde der Status quo bezüglich der Umsetzung der veröffentlichten Empfehlungen untersucht.

## Hintergrund

Gemeinsame Empfehlungen von Fachverbänden sollen die Entscheidungsfindung von Ärztinnen und Ärzten in spezifischen klinischen Situationen unterstützen, um durch möglichst einheitliches Handeln auf Basis aktuellen Wissens sowohl die Versorgungsqualität zu verbessern als auch die Patientensicherheit zu erhöhen.

Die Kategorisierung nichtelektiver Eingriffe war bisher durch eine Vielzahl unterschiedlicher Vorgehensweisen geprägt, wie beispielsweise das Nutzen von Ampelsystemen, von Begriffs- (Notfall, eilig, dringlich) oder Zeitskalen [3]. Diese waren zwar in den einzelnen Krankenhäusern intern individuell zumeist fest etabliert, dennoch war ein übergeordnetes einheitlichen Vorgehen nicht erkennbar. Mit der gemeinsamen Empfehlung von Anästhesiologen, Chirurgen und OP-Managern liegt seit 2016 eine Handlungsanweisung vor, welche eine interdisziplinär vereinbarte Nomenklatur für die Klassifikation von Notfalloperationen mit einer definierten zeitlichen Dimension hinsichtlich deren Durchführung verbindet. Diese konsentierte Empfehlung ermöglicht damit erstmals ein einheitliches, standardisiertes Vorgehen aller Beteiligten und somit eine Vergleichbarkeit des Managements von Notfalloperationen über Krankenhäuser unterschiedlichster Versorgungsstufen hinweg.

Das Entwickeln und Verbreiten von Empfehlungen als Instrument der Wissensvermittlung führt jedoch nicht alleinig zur angestrebten Verbesserung der Behandlungsqualität von Patienten, vielmehr ist der Transfer in den klinischen Alltag unabdingbar [12]. Umso wichtiger erscheint in diesem Kontext eine Evaluation der Umsetzung veröffentlichter Empfehlungen. Das Ziel der vorliegenden Studie ist eine Standortbestimmung, die analysiert, wie die gemeinsamen Empfehlungen zur Klassifikation operativer Notfalleingriffe im klinischen Alltag angekommen sind, umgesetzt und bewertet werden.

## Methodik

Die Erhebung der Daten im Rahmen der vorliegenden multizentrischen Studie erfolgte mithilfe einer Online-Umfrage. In die Studie eingeschlossen wurden Krankenhäuser sowie Krankenhausverbünde, die über einen operativen Bereich, einschließlich Notfallversorgung, sowie eine Mindestanzahl von 200 Krankenhausbetten verfügten. Als Zielgruppe zur Beantwortung des standardisierten Fragebogens wurden OP-Manager und OP-Koordinatoren sowie sonstige OP-Verantwortliche mit organisatorischem Weisungsrecht für den operativen Bereich ihres Krankenhauses definiert.

Das Entwickeln des Fragebogens sowie die Durchführung und Auswertung der Umfrage erfolgten unter Beachtung der Anforderungen an wissenschaftliche Befragungen [1]. Zum Erstellen und zum Versenden des webbasierten, zweigeteilten Fragebogens nutzten wir die kommerzielle Online-Plattform UmfrageOnline des Anbieters enuvo GmbH (Zürich, Schweiz). Ein erster Fragenkomplex bezog sich auf die Anwendung der Notfallkategorien im klinischen Alltag, einschließlich einer Bewertung, inwiefern diese die OP-Koordination unterstützen. Weiterhin wurden Aussagen zur Dokumentation und zur Auswertung des Notfallaufkommens sowie zu Konzepten der Integration von Notfällen in die vorhandene OP-Kapazität erhoben. In einem zweiten Fragenkomplex wurden Charakteristika der teilnehmenden Kliniken sowie Eckpunkte der Organisationsstruktur des OP-Bereiches erfragt. Patientenbezogene Daten wurden nicht erhoben. Die Fragen wurden als geschlossene sowie halboffene Fragen gestellt. Antwortmöglichkeiten wurden hinsichtlich der Auswahl mit „Multiple Choice“ und möglichen Mehrfachnennungen sowie mit „Single Choice“ unter Angabe von nur einer Antwortmöglichkeit aus zwei oder mehreren Alternativen vorgegeben. Eine komplette Beantwortung des ersten Fragekomplexes war notwendig, da bei Auslassen einer Frage die weitere Bearbeitung automatisch verweigert wurde. Durch einen Pretest wurde der Online-Fragebogen vor Start der Umfrage auf dessen Eignung hinsichtlich Verständlichkeit, Plausibilität und Länge überprüft [11]. An dieser probeweisen Befragung nahmen 5 zur Zielgruppe gehörende Personen sowie der Leiter des Instituts für Medizinische Psychologie und Medizinische Soziologie an der Universitätsmedizin Rostock teil. Der vollständige Online-Fragebogen ist als Zusatzmaterial online verfügbar.

Die Auswahl der an der Studie teilnehmenden Krankenhäuser erfolgte auf Grundlage des Registers deutscher Krankenhäuser in der Version 2017 [14]. Das Krankenhausverzeichnis weist mit Stand vom 31.12.2017 93,0 % aller deutschen Krankenhäuser sowie 96,4 % aller in Deutschland verfügbaren Krankenhausbetten aus. Alle den Einschlusskriterien entsprechenden Kliniken wurden vorab telefonisch kontaktiert, um mit dem Fragebogen gezielt das OP-Management bzw. den jeweiligen OP-Verantwortlichen zu erreichen. Hierdurch sollte ein sowohl quantitativ als auch qualitativ hochwertiger Rücklauf der Fragebogen sichergestellt werden. In einem nächsten Schritt wurden die Teilnehmer im Zeitraum vom 04.06.2019 bis 03.09.2019 durch den Versand einer personalisierten E‑Mail zur webbasierten Umfrage auf der Internetplattform www.umfrageonline.com eingeladen. Bei ausbleibender Reaktion erinnerten wir die Teilnehmer maximal 2‑mal nach 2 bzw. 6 Wochen per E‑Mail an die Befragung. Um eine mögliche Stichprobenverzerrung infolge von Nichtteilnahmen („nonresponse bias“) zu verhindern, deren Ursache bereits in der Unkenntnis der Empfehlungen begründet ist, wurde auf diese weder in der E-Mail-Einladung noch im Begrüßungstext der Umfrage Bezug genommen (Zusatzmaterial online). Die Übermittlung der Antworten der Teilnehmer erfolgte anonym, d. h., eine Zuordnung der Antworten zum Absender war nicht möglich. Hierauf wurden die Teilnehmer explizit hingewiesen. Für die Teilnehmer bestand jedoch die Möglichkeit, auf eigenen Wunsch diese Anonymisierung durch Angabe der eigenen Kontaktdaten aufzuheben.

Die Online-Umfrage wurde im Deutschen Register für Klinische Studien unter der Nummer DRKS00017105 registriert; ethische oder berufsrechtliche Bedenken seitens der Ethikkommission der Medizinischen Fakultät der Universität Rostock bestanden nicht.

Die Auswertung der Antworten erfolgte deskriptiv unter Zuordnung der absoluten und prozentualen Ergebnisse zum jeweiligen Versorgungstyp. Um einen möglichen Einfluss klinikspezifischer Faktoren auf diese Ergebnisse darzustellen, wurden zusätzlich ausgewählte Antworten unter Anwendung des Chi-Quadrat-Tests auf Abhängigkeiten untersucht. Die statistische Analyse der Daten wurde unter Verwendung der Programme Microsoft® Excel für Mac (Version Microsoft Office, 2020, Microsoft®, Redmond, Washington, USA) sowie SPSS® (Statistics Version 27, 2020, IBM®, Amonk, New York, USA) durchgeführt.

## Ergebnisse

Von den 550 OP-Verantwortlichen, die mit der Bitte um Teilnahme an der Befragung angeschrieben wurden, beantworteten 274 die ihnen zugesandten Fragen. Dies entspricht einer Rücklaufquote von 49,8 %. 18 Fragebogen (6,5 %) mussten aufgrund verfehlter Einschlusskriterien sowie infolge unvollständiger Datenangaben von der Auswertung ausgeschlossen werden. Letztendlich wurden die Antworten von 256 Teilnehmern und somit von 46,5 % aller Befragten in die statistische Analyse einbezogen.

Es antworteten 94 (36,7 %) OP-Verantwortliche aus Häusern der Grund- und Regelversorgung, 95 (37,1 %) der Schwerpunkt- und Zentralversorgung, 37 (14,5 %) mit Maximalversorgungsauftrag (ohne Universitätskliniken) und 30 (11,7 %) aus Universitätskliniken. Einen Überblick über die strukturellen Eckdaten der teilnehmenden Einrichtungen gibt Tab. 1.

**Table 1:** 

**Versorgungsauftrag**	*Universitätsklinik*	*Maximalversorgung*	*Schwerpunktversorgung*	*Grund- und Regelversorgung*	
	30 (11,7 %)	37 (14,5 %)	95 (37,1 %)	94 (36,7 %)	
**Trägerschaft**	*Universitäten*	*Öffentlich*	*Konfessionell/freigemeinnützig*	*Privat*	*Andere*
	26 (10,1 %)	110 (43,0 %)	78 (30,5 %)	34 (13,3 %)	8 (3,1 %)
**Bettenverfügbarkeit (Anzahl)**	*>1000*	*500–1000*	*200–499*		
	45 (17,6 %)	84 (32,8 %)	127 (49,6 %)		
**OP-Verfügbarkeit**	*>30 Säle*	*20–30 Säle*	*10–19 Säle*	*<10 Säle*	
	23 (9,0 %)	23 (9,0 %)	74 (28,9 %)	136 (53,1 %)	
**Operative Fachdisziplinen**	*Allgemeinchirurgie*	*Traumatologie*	*Gynäkologie/Geburtshilfe*	*HNO/MKG/Augen*	*Urologie*
	252 (98,4 %)	245 (95,7 %)	218 (85,2 %)	182 (71,1 %)	175 (68,4 %)
**Form der Auswertung von OP-Kennzahlen**	*Internes Reporting*	*Reporting und Benchmarking*	*Kein Reporting*		
	108 (42,2 %)	130 (50,8 %)	18 (7,0 %)		
**Organisation des OP-Managements**	*Zuordnung zu Klinik/Abteilung*	*Stabstelle der Geschäftsführung*	*Nicht eigenständig*^a^	*Kein OP-Management*	
	88 (34,4 %)	130 (50,8 %)	31 (12,1 %)	7 (2,7 %)	
**Berufsgruppe der antwortenden Teilnehmer**	*Geschäftsführung*	*Arzt*	*Pflege*	*Andere*	*Keine Angabe*
	9 (3,5 %)	175 (68,4 %)	50 (19,5 %)	17 (6,6 %)	5 (2,0 %)

^a^Nicht eigenständig: einzelne Mitarbeitende, die nicht einer Organisationseinheit zugeordnet sind

Zur Beurteilung, ob die erhobenen Daten (Stichprobe, *n* = 256) die Verhältnisse der Grundgesamtheit (*n* = 550) ausreichend gut widerspiegeln, wurden beide Gruppen unter Verwendung des 2‑Stichproben-Kolmogorov-Smirnov-Tests hinsichtlich ihrer Übereinstimmung untersucht. Als Vergleichsparameter diente hierbei die Bettenanzahl der betrachteten Häuser. Im Ergebnis konnte gezeigt werden, dass sich die Verteilung der Krankenhäuser in der Stichprobe nicht signifikant von der in der Grundgesamtheit unterschied (*p* = 0,643) und die Stichprobe folglich als repräsentativ angenommen werden kann.

**Figure 1:**
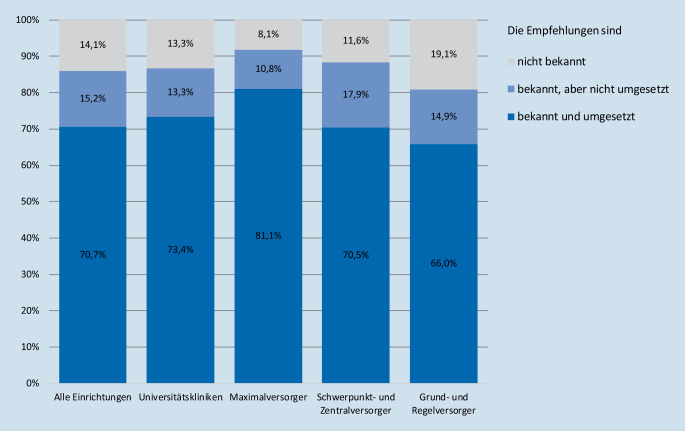

Die Empfehlungen zur Klassifikation von Notfalleingriffen und deren koordinative Umsetzung waren bundesweit 85,9 % der OP-Verantwortlichen (*n* = 220) bekannt. Der Bekanntheitsgrad erreichte in Häusern der Maximalversorgung (ohne Universitätskliniken) mit 91,9 % sowie an Häusern der Schwerpunktversorgung mit 88,4 % die höchsten Werte, in Häusern der Grund- und Regelversorgung mit 80,9 % den niedrigsten Wert. 181 der Befragten (70,7 %) gaben an, dass die Empfehlungen der Verbände in ihren Einrichtungen umgesetzt würden. In weiteren 15,2 % der Krankenhäuser waren die Empfehlungen zwar bekannt, fanden jedoch bisher im klinischen Alltag keine einheitliche Anwendung (Abb. 1). Wesentliche Instrumente der Implementierung der Notfallklassifikation waren die Verankerung im OP-Statut mit 84,5 % sowie die Integration in das Krankenhausinformationssystem mit 71,4 %.

**Figure 2:**
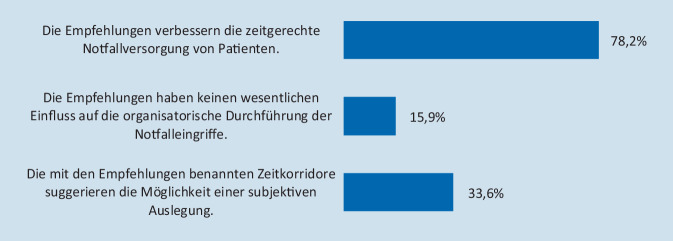

Allen Teilnehmern, die die Kenntnis der Notfallklassifikationen bestätigt hatten (*n* = 220), wurden im Folgenden Fragen zur Akzeptanz der im Glossar empfohlenen Vorgehensweise gestellt. Die Auffassung, die Empfehlungen verbessern die zeitgerechte Notfallversorgung von Patienten im klinischen Alltag, teilte eine Mehrheit von 78,2 % der Befragten (*n* = 172). 15,9 % der OP-Koordinatoren und OP-Manager (*n* = 35) sahen hingegen keinen wesentlichen Einfluss auf die organisatorische Umsetzung von Notfällen im Tagesgeschäft. Unabhängig von der Versorgungsstufe der Klinik waren 74 der Befragten (33,6 %) der Ansicht, dass die definierten Zeitintervalle zur Umsetzung von Notfällen gleichzeitig die Möglichkeit einer subjektiven Auslegung suggerieren könnten (Abb. 2). 80,1 % der OP-Verantwortlichen befürworteten zusätzliche hausinterne Empfehlungen. In Häusern, die diese bereits implementiert hatten (*n* = 100, 39,1 %), lag die positive Wahrnehmung dieser Maßnahme bei 95,0 %. Eine detaillierte Auflistung der Antworten stellt Tab. 2 dar.

**Table 2:** 

	Alle Einrichtungen	Universitätsklinik	Maximalversorgung	Schwerpunktversorgung	Grund- und Regelversorgung
**Kenntnis und Umsetzung der Empfehlungen**					
*Bekannt*	220 (85,9 %)	26 (86,7 %)	34 (91,9 %)	84 (88,4 %)	76 (80,9 %)
Bekannt und umgesetzt	181 (70,7 %)	22 (73,4 %)	30 (81,1 %)	67 (70,5 %)	62 (66,0 %)
Bekannt, aber nicht umgesetzt	39 (15,2 %)	4 (13,3 %)	4 (10,8 %)	17 (17,9 %)	14 (14,9 %)
*Nicht bekannt*	36 (14,1 %)	4 (13,3 %)	3 (8,1 %)	11 (11,6 %)	18 (19,1 %)
**Implementierung der Empfehlungen**					
*OP-Statut*	186 (84,5 %)	24 (92,3 %)	31 (91,2 %)	74 (88,1 %)	57 (75,0 %)
*KIS*^*a*^	157 (71,4 %)	23 (88,5 %)	26 (76,5 %)	63 (75,0 %)	45 (59,2 %)
**Einfluss der Empfehlungen auf die zeitgerechte Notfallversorgung von Patienten**^**b**^					
*Verbessert die Versorgung*	172 (78,2 %)	20 (76,9 %)	27 (79,4 %)	72 (85,7 %)	53 (69,7 %)
*Kein Einfluss*	35 (15,9 %)	6 (23,1 %)	7 (20,6 %)	10 (11,9 %)	12 (15,8 %)
*Subjektivität möglich*	74 (33,6 %)	10 (38,5 %)	10 (29,4 %)	27 (32,1 %)	27 (35,5 %)
**Beurteilung von zusätzlichen hausinternen Empfehlungen**					
*Hilfreich*	205 (80,1 %)	20 (66,7 %)	29 (78,4 %)	75 (78,9 %)	81 (86,2 %)
*Nicht hilfreich*	51 (19,9 %)	10 (33,3 %)	8 (21,6 %)	20 (21,1 %)	13 (13,8 %)
*Implementiert*	100 (39,1 %)	8 (26,7 %)	16 (43,2 %)	37 (38,9 %)	39 (41,5 %)

^a^Krankenhausinformationssystem

^b^Mehrfachantworten möglich

**Figure 3:**
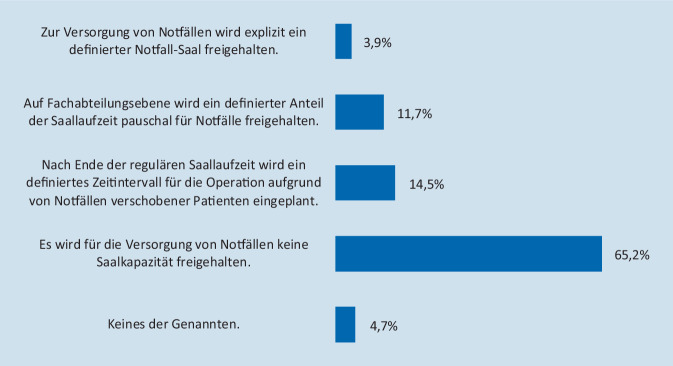

Das hauseigene Notfallaufkommen war 62,9 % der OP-Verantwortlichen bekannt; 47,3 % der Kliniken werteten dieses regelmäßig aus. Für die Versorgung von Notfällen wurde in 65,2 % der deutschen Krankenhäuser keine definierte Saalkapazität freigehalten. 3,9 % der Häuser antworteten, dass sie zur Versorgung von Notfällen explizit einen definierten Notfallsaal mit einem frei verfügbaren Team vorhalten, welcher in der Kernbetriebszeit interdisziplinär für die operative Versorgung von Notfällen genutzt werden kann. 26,2 % der Kliniken berücksichtigten die für Notfalleingriffe notwendige Kapazität bei der Planung des OP-Programms bzw. bei der Festlegung von Saallaufzeiten (Abb. 3). Auf das konzeptionelle Vorgehen bei der Bereitstellung von Notfallkapazität hatten weder der Versorgungstyp (im Vergleich zwischen Häusern der Maximalversorgung, einschließlich Universitätsklinika und Häusern der Schwerpunkt‑, Regel- und Grundversorgung, *p* = 0,42) noch das Vorhalten einer geburtshilflichen Abteilung (*p* = 0,98), die Gesamtzahl der betriebenen OP-Säle (*p* = 0,41) oder ein regelmäßiges Reporting des Notfallaufkommens (*p* = 0,11) einen signifikanten Einfluss. Die detaillierten Ergebnisse der Befragung einschließlich Unterteilung nach Versorgungstypen sind in Tab. 3 zusammengefasst.

**Table 3:** 

	Alle Einrichtungen	Universitätsklinik	Maximalversorgung	Schwerpunktversorgung	Grund- und Regelversorgung
**Dokumentation des hausinternen Notfallaufkommens**					
*Ja*	149 (58,2 %)	24 (80,0 %)	26 (70,3 %)	56 (58,9 %)	43 (45,7 %)
*Nein*	107 (41,8 %)	6 (20,0 %)	11 (29,7 %)	39 (41,1 %)	51 (54,3 %)
**Kenntnis des durchschnittlichen Notfallaufkommens**					
*Ja*	161 (62,9 %)	23 (76,7 %)	25 (67,6 %)	69 (72,6 %)	44 (46,8 %)
*Nein*	95 (37,1 %)	7 (23,3 %)	12 (32,4 %)	26 (27,4 %)	50 (53,2 %)
**Hausinternes Reporting des Notfallaufkommens**					
*Ja*	121 (47,3 %)	20 (66,7 %)	25 (67,6 %)	48 (50,5 %)	28 (29,8 %)
*Nein*	135 (52,7 %)	10 (33,3 %)	12 (32,4 %)	47 (49,5 %)	66 (70,2 %)
**Vorhaltung von OP-Kapazität zur Notfallversorgung**					
*Notfallsaal*	10 (3,9 %)	1 (3,3 %)	3 (8,1 %)	5 (5,3 %)	1 (1,1 %)
*Zeitanteil 1*	30 (11,7 %)	4 (13,3 %)	1 (2,7 %)	9 (9,5 %)	16 (17,0 %)
*Zeitanteil 2*	37 (14,5 %)	6 (20,0 %)	4 (10,8 %)	16 (16,8 %)	11 (11,7 %)
*Keine*	167 (65,2 %)	17 (56,7 %)	28 (75,7 %)	63 (66,3 %)	59 (62,8 %)
*Sonstige*	12 (4,7 %)	2 (6,7 %)	1 (2,7 %)	2 (2,1 %)	7 (7,4 %)

*Zeitanteil 1*: Auf Fachabteilungsebene wird bei Planung des OP-Programms ein definierter Anteil der regulären Saallaufzeit pauschal für die Versorgung von Notfällen freigehalten

*Zeitanteil 2*: Nach Ende der regulären Saallaufzeit wird ein definiertes Zeitintervall für die Operation elektiver Patienten, die infolge der Versorgung von Notfällen zeitlich verschoben werden mussten, eingeplant

## Diskussion

Die vorliegende Arbeit gibt erstmals einen Überblick, wie die Empfehlungen der Fachverbände zur Klassifikation von Notfalleingriffen an deutschen Kliniken umgesetzt werden (Infobox 1). Darüber hinaus bietet sie Anhaltspunkte, wie Maßnahmen und Konzepte zur zeitgerechten Durchführung von Notfalloperationen von den OP-Verantwortlichen genutzt bzw. beurteilt werden.

Die Empfehlungen von BDA/DGAI, BDC/DGCH und VOPM zur Klassifikation von Notfällen, erschienen im Jahr 2016, kommen in Krankenhäusern aller Versorgungsstufen in Deutschland zur Anwendung. Zweieinhalb Jahre nach der Veröffentlichung sind die fachübergreifenden Empfehlungen in hohem Maße sowohl bekannt (85,9 % der Antwortenden) als auch bereits im klinischen Alltag umgesetzt (70,7 % der teilnehmenden Krankenhäuser). Dieser Durchdringungsgrad ist vergleichbar mit der Verankerung anderer elementarer Handlungsempfehlungen in der Anästhesiologie. So waren beispielsweise die Empfehlungen zur präoperativen Risikoevaluation erwachsener Patienten vor elektiven, nichtkardiochirurgischen Eingriffen zwei Jahre nach deren Veröffentlichung 84,2 % der Anästhesisten bekannt und in 71,4 % vollständig oder teilweise implementiert [6]. Bei den Empfehlungen zur verkürzten präoperativen Nüchternheit liegen diese Zahlen mit 90 % bzw. 75 % in einem vergleichbar hohen Bereich [8]. Die Aufnahme entsprechender Regelungen zur zeitlichen Koordination von Notfalloperationen in das eigene OP-Statut sowie die Implementierung der verschiedenen Dringlichkeitsstufen in das hausinterne Krankenhausinformationssystem wurden von einer großen Mehrheit bereits realisiert. Von Bedeutung erscheint insbesondere der letzte Punkt, wird damit doch sowohl den Anforderungen an eine hohe interdisziplinäre und interprofessionelle Transparenz als auch einer zeitgenauen Dokumentation unter medikolegalen Aspekten Rechnung getragen. In der aktualisierten, überarbeiteten Version 2020 [2] wurde aus vorgenannten Gründen eine Revision der Klassifikation der Dringlichkeit von Operationen u. a. mit dem Ziel vorgenommen, durch eine vereinheitlichte N‑Terminologie zu einer verbesserten visuellen Übersichtlichkeit im Krankenhausinformationssystem beizutragen. Eingriffe der Kategorie „Dringlich“ werden nunmehr mit „N4“ verschlüsselt, elektive Operationen mit „N5“.

Die Empfehlungen werden von der großen Mehrheit der OP-Verantwortlichen als hilfreich bei der Koordination des Notfallgeschehens erachtet. Eine deutliche Dreiviertelmehrheit ist der Ansicht, dass die Empfehlungen die zeitgerechte Versorgung von Notfallpatienten verbessern. Dies kann als eindeutiger Hinweis auf eine breite Akzeptanz der Empfehlungen im klinischen Alltag gewertet werden, umso mehr vor dem Hintergrund der im Vergleich zu Leitlinien eher geringen Verbindlichkeit von Empfehlungen für das eigene Handeln. Jeder dritte Antwortende ist allerdings gleichzeitig der Ansicht, dass die definierten Zeitintervalle zur Umsetzung der Notfälle die Möglichkeit einer subjektiven Auslegung suggerieren könnten. Dies widerspricht zwar nicht der erfolgreichen Verankerung der Notfallklassifikationen, verdeutlicht jedoch eine Problematik, die mit der auf einer rein zeitlichen Dimension basierenden Unterteilung möglicherweise einhergeht. Während die Vorgabe klarer Zeitintervalle bei der koordinativen Umsetzung hilfreich ist, eröffnet sie bei der Klassifikation von Notfalleingriffen evtl. die Option einer ebenfalls eher organisatorischen, subjektiv begründeten OP-Meldung, deren Fokus hauptsächlich auf dem zu erwartenden Zeitpunkt der Durchführung liegt. Aufgrund der Verantwortlichkeit des Operateurs für die medizinische Indikationsstellung ist das OP-Management an die entsprechende Umsetzung gebunden, auch wenn der entsprechende Eingriff im Vergleich zu Erfahrungswerten eher keine typische Klassifikation aufweisen sollte. Vor diesem Hintergrund befürworten über 80 % der Antwortenden zusätzliche hausinterne Empfehlungen. In Häusern, die aktuell mit diesen bereits arbeiten, liegt der Zustimmungsgrad sogar bei 95,0 %. Ein Beispiel für eine derartige ergänzende, indikationsbezogene Liste häufig auftretender Notfälle findet sich bei Wienströer [17]. Aus Sicht der Autoren ist es durchaus überlegenswert, eine Aufstellung typischer Indikationsstellungen für die deutschlandweit häufigsten Notfalloperationen in Abstimmung zwischen den Fachverbänden zu erarbeiten, welche ergänzend zu den aktuellen Empfehlungen die Koordination von Notfalleingriffen unterstützen könnte.

Die Frage nach der OP-Kapazität, die im Tagesgeschäft für Notfallkapazitäten freigehalten werden muss, wird seit Jahren kontrovers diskutiert. Während eine Erhebung des Deutschen Krankenhausinstituts [5] zeigt, dass Kliniken in Deutschland im Jahr 2015 durchschnittlich einen Notfallsaal explizit zur operativen Versorgung von Notfällen vorgehalten haben, plädieren Schüpfer et al. dafür, dass die Häufigkeit von Notfalloperationen bei der OP-Kapazitätsplanung nicht überbewertet werden sollte [13]. Studien, die die Effizienz einzelner Modelle untersuchten, weisen zudem sehr gegensätzliche Aussagen auf. Während van Veen-Berkx et al. [16] mit Blick auf eine höhere Saalauslastung bei gleichzeitig weniger OP-Absagen einen definierten Notfallsaal favorisieren, unterstützen andere Publikationen [7, 15, 18] das zeitgerechte Verteilen der Notfallpatienten über alle regulär laufenden OP. Vor diesem Hintergrund liefern die Ergebnisse der vorliegenden Studie wichtige, teils neue Erkenntnisse. Einerseits kann festgestellt werden, dass entgegen bisherigen Annahmen das Vorhalten eines expliziten Notfallsaals im Jahr 2019 nur noch die Ausnahme (in 3,9 % der Kliniken) zu sein scheint. Die sich hieraus ergebene Vermutung, dass der klassische Notfallsaal durch andere Konzepte zur Integration von Notfallkapazität abgelöst wurde, konnte andererseits nicht bestätigt werden. Vielmehr geben fast zwei Drittel der Kliniken an, für die Notfallversorgung keine Kapazität frei zu halten; nur ca. jede vierte Klinik bezieht diese in ihre Planungen ein. In diesem Kontext stellt sich dem OP-Verantwortlichen in der klinischen Realität letztendlich folgende Frage: Wie kann man ausreichend OP-Kapazität frei halten, um das Notfallgeschehen zeitgerecht abarbeiten zu können, und gleichzeitig verhindern, dass durch einen nichtgenutzten OP Leerkosten bis zu 800 €/h entstehen [9]? Hier empfehlen sich hausinterne Vereinbarungen, die beispielsweise für vitale Notfälle in der Kernbetriebszeit das Freihalten eines OP bzw. die Nutzung des nächsten frei werdenden Saales erst bei konkretem Verdacht beinhalten [13]. Ebenfalls sollten im OP-Statut transparente Regelungen verankert werden, die den Umgang mit elektiven, infolge von Notfällen verschobenen Programmpunkten beschreiben. Eine mögliche Vorgehensweise besteht in der Berücksichtigung von anteiliger OP-Kapazität für Notfälle bereits in der OP-Planung [3]. Eine Möglichkeit sieht das pauschale Einbehalten einer definierten prozentualen OP-Kapazität am Ende der regulären Saallaufzeit vor, insbesondere bei Fachabteilungen mit einem hohen Notfallaufkommen. Alternativ kann ein definiertes Zeitintervall nach Ende der Regelsaallaufzeit eingeplant werden, in welchem verschobene elektive Eingriffe, ggf. interdisziplinär, taggleich durchgeführt werden können. Grundvoraussetzungen für eine valide prospektive Berücksichtigung des Notfallaufkommens in der OP-Planung sind in jedem Fall eine differenzierte Dokumentation und Auswertung aller umgesetzten operativen Notfälle [10]. Die Ergebnisse der vorliegenden Arbeit zeigen jedoch in diesem Bereich bei knapp der Hälfte der Kliniken noch einen Nachholbedarf. Dies ist umso erstaunlicher, da in 93,0 % der teilnehmenden Kliniken ein internes Reporting der wichtigsten OP-Kennzahlen, zum überwiegenden Teil mit Teilnahme an einem Benchmark-Programm, bereits etabliert ist.

Durch die Befragung der OP-Verantwortlichen zum Umgang mit den Empfehlungen der Fachverbände konnte erstmals gezeigt werden, wie diese im Klinikalltag genutzt und bewertet werden. Die Umfrage zielt im Wesentlichen auf die Erfahrung und Einschätzung von OP-Managern und OP-Koordinatoren als die für den organisatorischen Prozess hauptsächlich Verantwortlichen ab. Eine Limitation der vorliegenden Studie besteht demzufolge in der Tatsache, dass keine Aussagen zum spezifischen Kenntnisstand bzw. zur Beurteilung der anderen an der Umsetzung beteiligten Berufsgruppen möglich sind. Eine zusätzliche Erhebung, beispielsweise unter Chirurgen, könnte indes die dargestellten Ergebnisse durch eine fachspezifische Sicht differenziert ergänzen. Des Weiteren sollten detaillierte Aussagen zur Häufigkeit von Notfalloperationen und deren zeitlichen Realisierung, insbesondere in Abhängigkeit vorhandener Ressourcen, Ziel weiterführender Untersuchungen sein.

### Infobox 1 Empfohlene Maßnahmen zur erfolgreichen Implementierung der Notfallklassifikationen in der klinischen Praxis

- Aufnahme der Notfallklassifikationen und davon abgeleiteter Regelungen zur zeitlichen Koordination von Notfalloperationen in das eigene OP-Statut
- Verankerung transparenter Regeln im OP-Statut, die den Umgang mit elektiven, infolge von Notfällen verschobenen Programmpunkten beschreiben
- Durchführung interdisziplinärer und interprofessioneller Fortbildungsmaßnahmen zu Inhalten und zur Anwendung der Notfallklassifikationen
- Implementierung der verschiedenen Dringlichkeitsstufen in das hausinterne Krankenhausinformationssystem bzw. OP-Dokumentationssystem
- Differenzierte Dokumentation und Auswertung aller umgesetzten operativen Notfälle sowie Integration der analysierten OP-Kennzahlen in das hausinterne Berichtswesen
- Erarbeiten hauseigener Konzepte zur Vorhaltung von Notfallkapazitäten auf Basis einer Bedarfsrechnung und im Konsens zwischen den Fachabteilungen, beispielsweise durch
  - das pauschale Einbehalten einer definierten prozentualen OP-Kapazität am Ende der regulären Saallaufzeit
  - das Einplanen eines definierten Zeitintervalls nach Ende der Regelsaallaufzeit, in welchem verschobene elektive Eingriffe, ggf. interdisziplinär, taggleich durchgeführt werden können
- Bei Bedarf hausinterne Ergänzung der Notfallklassifikationen durch fachabteilungsspezifische Empfehlungen zu den häufigsten Notfallindikationen

## Fazit für die Praxis

Die Empfehlungen zur Klassifikation von Notfalleingriffen, jetzt in der aktualisierten Version 2020 vorliegend, sind ein wichtiges, allgemein anerkanntes Steuerungselement im Rahmen der OP-Koordination. Sie erleichtern die fächerübergreifende Organisation und Kommunikation bei der Integration von Notfällen und verbessern nach Meinung der meisten OP-Verantwortlichen die zeitgerechte Versorgung der Patienten. Neben der Verankerung im OP-Statut kann insbesondere die Implementierung der Notfallkategorien im Krankenhausinformationssystem die Umsetzung unterstützen. Die Kenntnis der wichtigsten Kennzahlen des hauseigenen Notfallaufkommens stellt eine wichtige Grundlage für die Steuerung des Gesamt-OP einschließlich der Notfallintegration dar. Das Vorhalten eines eigenen Notfallsaales bleibt entgegen der bisherigen Meinung zunehmend die Ausnahme. Ein an die Rahmenbedingungen vor Ort adaptiertes Konzept zur Vorhaltung von Notfallkapazitäten im Tagesbetrieb sollte hausintern auf Basis einer Bedarfsrechnung und im Konsens zwischen den Fachabteilungen etabliert werden.

## Supplementary Information




