# Supplementary material for: Implementation of emergency classifications—Where do we stand? Results of a nationwide survey
Source: Anaesthesist. 2021 May 18;70(12):1003–10. [Article in German] doi: 10.1007/s00101-021-00971-2 (PMC8639562; doi:10.1007/s00101-021-00971-2)
Supplement: Supplementary file 1 [file 101_2021_971_MOESM1_ESM.pdf]

Zusatzmaterial zum Beitrag „Der Umgang mit Notfallklassifikationen – Wo stehen wir? Ergebnisse einer deutschlandweiten Umfrage“ von Brosin, A; Kropp, P; Reuter, DA und Janda M (2021) in *Der Anaesthesist*.
Beitrag und Zusatzmaterial stehen Ihnen auf www.springermedizin.de zur Verfügung. Bitte geben Sie dort den Beitragstitel in die Suche ein.

Fragebogen zum Management von Notfall-Eingriffen im OP

Sehr geehrte Kollegin,
sehr geehrte Kollege,

das Management von Notfalleingriffen im OP stellt eine wesentliche Herausforderung unserer täglichen Arbeit dar. Wir alle sind daran interessiert, diese Abläufe bestmöglich zu organisieren, sehen uns hierbei jedoch immer wieder unterschiedlichsten Herausforderungen gegenüber.

Die nachfolgende Erhebung befasst sich mit der medizinischen Dringlichkeit operativer Notfälle und der damit einhergehenden koordinativen Reaktion im operativen Tagesgeschäft.

Die anonyme Umfrage umfasst 11 Fragen und dauert nicht länger als 5-7 Minuten.
Es gibt keine richtigen oder falschen Antworten – was zählt, ist Ihre Meinung. Wir freuen uns auf Ihr Feedback.

Ist Ihnen die nachfolgend dargestellte, von BDA/DGAI, BDC/DGCH und VOPM vereinheitlichte Notfall-klassifikation bekannt?

○ ja

○ nein

Notfallklassifikation	
N0	**Eingriff höchster Dringlichkeit**<br>Bem.: Vorschlag zur Koordination innerhalb der Saalöffnungszeiten: Operation sofort, ggf. unmittelbar am Aufenthaltsort des Patienten (z.B. Schockraum, Intensivstation, Kreißsaal).
N1	**Sehr hohe Dringlichkeit**<br>Bem.: Vorschlag zur Koordination innerhalb der Saalöffnungszeiten: OP auf dem nächsten freien geeigneten Tisch, unabhängig von der Fachdisziplin.
N2	**OP-Beginn ≤6 h nach Meldung**<br>Bem.: Vorschlag zur Koordination innerhalb der Saalöffnungszeiten: OP auf dem nächsten freien Tisch der eigenen Fachdisziplin. Soweit möglich, Abwarten der Nüchternheit, unfallchirurgische Primärversorgung.
N3	**OP am Ende des Elektivprogramms im Rahmen des Notfallmanagements**
dringlich:	**OP innerhalb 12-24h**<br>Bem.: Vorschlag zur Koordination innerhalb der Saalöffnungszeiten: Einpflegen in das OP-Programm des Folgetags (auch nach abgeschlossener OP-Planung unter Wahrung der Stabilität des 1. Punktes).

Bauer M. et al.: Glossar perioperativer Prozesszeiten und Kennzahlen – Eine gemeinsame Empfehlung von BDA / DGAI, BDC / DGHC und VOPM. Anästh. Intenivmed. 2016; 57: 669-683

Wurden die Empfehlungen zur Notfallklassifikation in Ihrem Haus bereits umgesetzt?

○ ja

○ nein

Sind die vereinheitlichen Definitionen der medizinischen Dringlichkeit sowie deren koordinativer Umgang mit Notfalloperationen im OP-Statut festgeschrieben?

○ ja

○ nein

Ist die Klassifikation der Notfälle in Ihrem Krankenhausinformationssytem integriert?

○ ja

○ nein

Erachten Sie die Empfehlungen zum koordinativen Umgang mit Notfalloperationen als hilfreichen Leitfaden bei der Umsetzung des Notfallaufkommens? (Maximal 2 Antworten möglich)

○ Die Empfehlungen verbessern die zeitgerechte Notfallversorgung von Patienten

○ Die Empfehlungen haben keinen wesentlichen Einfluss auf die organisatorische Durchführung der Notfalleingriffe.

○ Die mit den Empfehlungen benannten Zeitkorridore suggerieren die Möglichkeit einer subjektiven Auslegung.

Findet eine regelmäßige Dokumentation des Notfallaufkommens in Ihrem OP statt?

○ ja

○ nein

Ist Ihnen das durchschnittliche Notfallaufkommen mit Versorgungspflicht innerhalb der Kernbetriebszeit Ihres OP' s bekannt?

○ ja

○ nein

Werten Sie das Notfallaufkommen des operativen Bereichs Ihres Hauses im Rahmen regelmäßiger Reportings aus?

○ ja

○ nein

Gibt es in Ihrem Haus interne Empfehlungen für die Klassifikation der am häufigsten auftretenden Notfalleingriffe?

○ ja

○ nein

Erachten Sie eine (mögliche) Implementierung derartiger Empfehlungen als hilfreich?

○ ja

○ nein

Für die Sicherstellung der bedarfsgerechten Verfügbarkeit von Saalkapazitäten für die Versorgung von Notfällen existieren verschiedene, nachfolgend beschriebene Konzepte.

Welchem der beschriebenen Konzepte entspricht das Vorgehen in Ihrer Einrichtung am ehesten?

○ Zur Versorgung von Notfällen wird explizit ein definierter Notfall-Saal einschließlich frei verfügbarem Team vorgehalten, der in der Kernbetriebszeit interdisziplinär für Notfalleingriffe genutzt werden kann.

○ Auf Fachabteilungsebene wird bei Planung des OP-Programms ein definierter Anteil der regulären Saallaufzeit pauschal für die Versorgung von Notfällen freigehalten.

○ Nach Ende der regulären Saallaufzeit wird ein definiertes Zeitintervall für Operationen elektiver Patienten, die infolge der Versorgung von Notfällen zeitlich verschoben werden mussten, eingeplant.

○ Es wird für die Versorgung von Notfällen keine Saalkapazität freigehalten.

○ Keinem der genannten

Welchem Versorgungsauftrag hat Ihr Krankenhaus / Verbund?

○ Universitätsklinikum

○ Maximalversorgung (ohne Universitätsklinika)

○ Schwerpunkt- / Zentralversorgung

○ Grund- und Regelversorgung

○ Übrige [ ]

In welcher Trägerschaft befindet sich Ihr Krankenhaus / Verbund?

○ Gebietskörperschaft (öffentlich-rechtlicher Träger, z. B. kommunal)

○ Universitätsklinikum

○ Konfessioneller Träger (freigemeinnütziger Träger)

○ Privater Träger

○ Sonstiger Träger

Über wie viele Betten verfügt Ihr Krankenhaus / Verbund?

○ < 200

○ 200 – 499

○ 500 – 1000

○ > 1000

Wie viele OP-Säle insgesamt werden in Ihrem Krankenhaus / Verbund werktäglich betrieben?

○ < 10

○ 10 bis 19

○ 20 bis 30

○ > 30

Welche operativen Fachabteilungen umfasst Ihr Krankenhaus / Verbund?
(Mehrfachantworten möglich)

○ Allgemeinchirurgie

○ Unfallchirurgie

○ Herzchirurgie

○ Gynäkologie/Geburtshilfe

○ Kinderchirurgie

○ Kopf/Hals (HNO/MKG/Augen)

○ Neurochirurgie

○ Urologie

○ Sonstige Fachabteilung

Wie ist das OP-Management Ihres Krankenhauses / Verbundes organisiert?

○ **Eigenständig**: Organisatorisch fest einer Klinik/Abteilung (z.B. Anästhesie, Chirurgie) oder der Geschäftsführung zugeordnet

Eigenständiges OP-Management

○ Organisatorisch fest einer Klinik/Abteilung zugeordnet (z.B. Anästhesie, Chirurgie)

○ Organisatorisch eigenständiger Bereich (z.B. als Stabsstelle der Geschäftsführung)

○ **Nicht eigenständig**: Mitarbeitern des ärztlichen Dienstes oder der Pflege zugeordnet (einzeln oder wechselnd im Team)

Nicht eigenständiges OP-Management

○ Team-Lösung (z.B. berufsgruppen- oder abteilungsübergreifend, wechselnde Verantwortlichkeiten innerhalb einer Gruppe)

○ Arzt Anästhesie

○ Arzt operative Fachabteilung

○ Anästhesie-Pflege

○ OP-Pflege

○ Kein OP-Management

Welche Form der Auswertung von OP-Kennzahlen nutzt Ihr Krankenhaus / Verbund?

○ Reporting / Internes Berichtswesen (Auswertung nur auf das eigene Krankenhaus bezogen ohne Vergleichsmöglichkeit mit anderen Krankenhäusern)

○ Reporting / Internes Berichtswesen sowie Teilnahme an einem Benchmarkprogramm (Vergleichsmöglichkeit mit anderen Krankenhäusern)

○ Kein Reporting / kein Benchmarking

Dieser Fragebogen wurde beantwortet von einer Mitarbeiterin / einem Mitarbeiter

○ des Ärztlichen Dienstes

○ der Berufsgruppe der Pflege

○ der Geschäftsführung

○ einer anderen Berufsgruppe ______

Ihre Kontaktdaten:

Bei Interesse an den Ergebnissen dieser Umfrage können Sie uns gerne Ihre Kontaktdaten hinterlassen. Die Angabe erfolgt selbstverständlich freiwillig, zumal die Anonymität der Befragung hierdurch unter Umständen aufgehoben wird.

Name ______

Email-Adresse ______
